# Comparative genomics analysis of the multidrug-resistant *Aeromonas hydrophila* MX16A providing insights into antibiotic resistance genes

**DOI:** 10.3389/fcimb.2022.1042350

**Published:** 2022-11-03

**Authors:** Yuxin Guo, Chenxi Zeng, Chenjie Ma, Hongjiao Cai, Xinglong Jiang, Shaowei Zhai, Xiaojin Xu, Mao Lin

**Affiliations:** ^1^ Fisheries College, Engineering Research Center of the Modern Technology for Eel Industry, Ministry of Education, Jimei University, Xiamen, Fujian, China; ^2^ Key Laboratory of Healthy Mariculture for the East China Sea, Ministry of Agriculture and Rural Affairs, Xiamen, Fujian, China

**Keywords:** *Aeromonas hydrophila*, complete genome sequencing, comparative genomics, multidrug resistance, drug resistance mechanism

## Abstract

In this paper, the whole genome of the multidrug-resistant *Aeromonas hydrophila* MX16A was comprehensively analyzed and compared after sequencing by PacBio RS II. To shed light on the drug resistance mechanism of *A. hydrophila* MX16A, a Kirby-Bauer disk diffusion method was used to assess the phenotypic drug susceptibility. Importantly, resistance against β-lactam, sulfonamides, rifamycins, macrolides, tetracyclines and chloramphenicols was largely consistent with the prediction analysis results of drug resistance genes in the CARD database. The varied types of resistance genes identified from *A. hydrophila* MX16A revealed multiple resistance mechanisms, including enzyme inactivation, gene mutation and active effusion. The publicly available complete genomes of 35 *Aeromonas hydrophila* strains on NCBI, including MX16A, were downloaded for genomic comparison and analysis. The analysis of 33 genomes with ANI greater than 95% showed that the pan-genome consisted of 9556 genes, and the core genes converged to 3485 genes. In summary, the obtained results showed that *A. hydrophila* exhibited a great genomic diversity as well as diverse metabolic function and it is believed that frequent exchanges between strains lead to the horizontal transfer of drug resistance genes.

## Introduction

Occurrences of multi-drug resistance (MDR) have become increasingly frequent in recent decades, complicating the treatment of certain diseases caused by microorganisms in humans (Williams and Bardsley, 1999; Thomas et al., 2016; Boucher et al., 2017). Multidrug-resistant bacteria (MDRB) could render a large number of antibiotic drugs ineffective, leading to severe public health concerns and affecting virtually all population groups around the world (Lautenbach and Fishman, 1999).The antibiotic-rich environment promotes horizontal gene transfer between bacteria, increasing the drug resistance of bacteria and causing a great risk to the public (Skwor et al., 2020).Furthermore, with constant evolution of MDRB, food production and safety may also be affected.

*Aeromonas* spp., a rod-shape and facultative anaerobic bacteria species, is widely distributed in natural environments, particularly in aquatic media (Chauret et al., 2001; Scoaris et al., 2008; Araujo et al., 1991; Rafei et al., 2018). The mesophilic species exhibits optimal growth at 35–37 °C and is involved in a variety of human infections whereas sychrophilic species can be grown at lower temperatures of 22–25 °C and frequently result in nonmotile diseases in fish (Janda and Abbott, 2010). This species of gram-negative bacteria, *A. hydrophila* being one representative, is commonly associated with a variety of diseases including septicaemia, gastroenteritis, and wound infections frequently affecting aquatic animals, terrestrial animals, and humans (Adamski et al., 2006; Parker and Shaw, 2011; Igbinosa et al., 2012; Rutteman, 2017).

From 1977 to 2016, three generations of various types of sequencing technologies have been developed. The second and third generation sequencing technologies referred commonly to as next generation sequencing technology, has evolved significantly with increase in sequencing speed, decrease in sequencing cost, since its inception in 2004. Currently, the third-generation sequencing platforms include Helicos™ Genetic Analysis System by SeqLL, SMRT Sequencing by Pacific Biosciences, Nanopore sequencing by Oxford Nanopore, to name a few (Ambardar et al., 2016). PacBio’s DNA sequencing has been used for detailed genomic investigations of bacterial isolates. Whole genome sequencing of bacteria has become an important tool for understanding and analyzing bacterial evolution. The complete understanding of the antibiotic resistance mechanisms of MDR *A. hydrophila* is crucial for the further development of therapeutic agents. We therefore conducted PacBio’s single molecule real time (SMRT™) DNA sequencing and the preliminary analysis of the MDRB strain *A. hydrophila* MX16A. Our results highlight resistance-related genes to aid in the understanding of genetic mechanisms of drug resistance of *A. hydrophila* and the preliminary prediction of virulence factors present in the genome. We further conducted a comparative genomic analysis of MX16A and reference *A. hydrophila* strains to generate the pan-core genome in order to understand the exclusive and shared genes and characteristics of the species of *A. hydrophila*. The results obtained herein will mine and develop the value of the existing reference genomes, and gain complete variation information of the entire species.

## Materials and methods

### Bacterial strain and antimicrobial susceptibility test

*A. hydrophila* strain MX16A was isolated from Jiulong River, Fujian, China (Lin et al., 2015), and cultured with Luria-Bertani broth (Hopebio, Qingdao, China) at 30 °C in a shaking incubator for 24 h. In previous experiments, MX16A was found to be insensitive to a variety of antibiotics, and contain various resistance genes and virulence genes detected by PCR.The toxicity tests showed that the median lethal concentration (LD50) of *A. hydrophila* MX16A to zebrafish was 1.6×10^6^ CFU/fish, indicating that MX16A had pathogenicity to aquatic animals. Antimicrobial susceptibility testing was performed using the Kirby-Bauer disk diffusion method (OXOID, UK). All results were determined by the Clinical and Laboratory Standards Institude (CLSI) guidelines (M100-S23).

### DNA extraction and 16S rRNA gene sequencing

Bacterial genomic DNA was extracted using the TIANamp Bacteria DNA Kit (Tiangen Biotech, Beijing, China) according to the manufacturer’s instructions. The strain identity was confirmed by 16S rRNA gene sequencing using specific primers (27F: 5’-AGAGTTTGATCCTGGCTCA G-3’; 1492R: 5’-GGTTACCTTGTTACGACTT-3’). The 16S rRNA sequence results from *A. hydrophila* MX16A were deposited into the GenBank Database with accession number KJ806394.1 (https://www.ncbi.nlm.nih.gov/search/all/?term=KJ806394.1).

### Library preparation and whole genome sequencing

**Figure 1:**
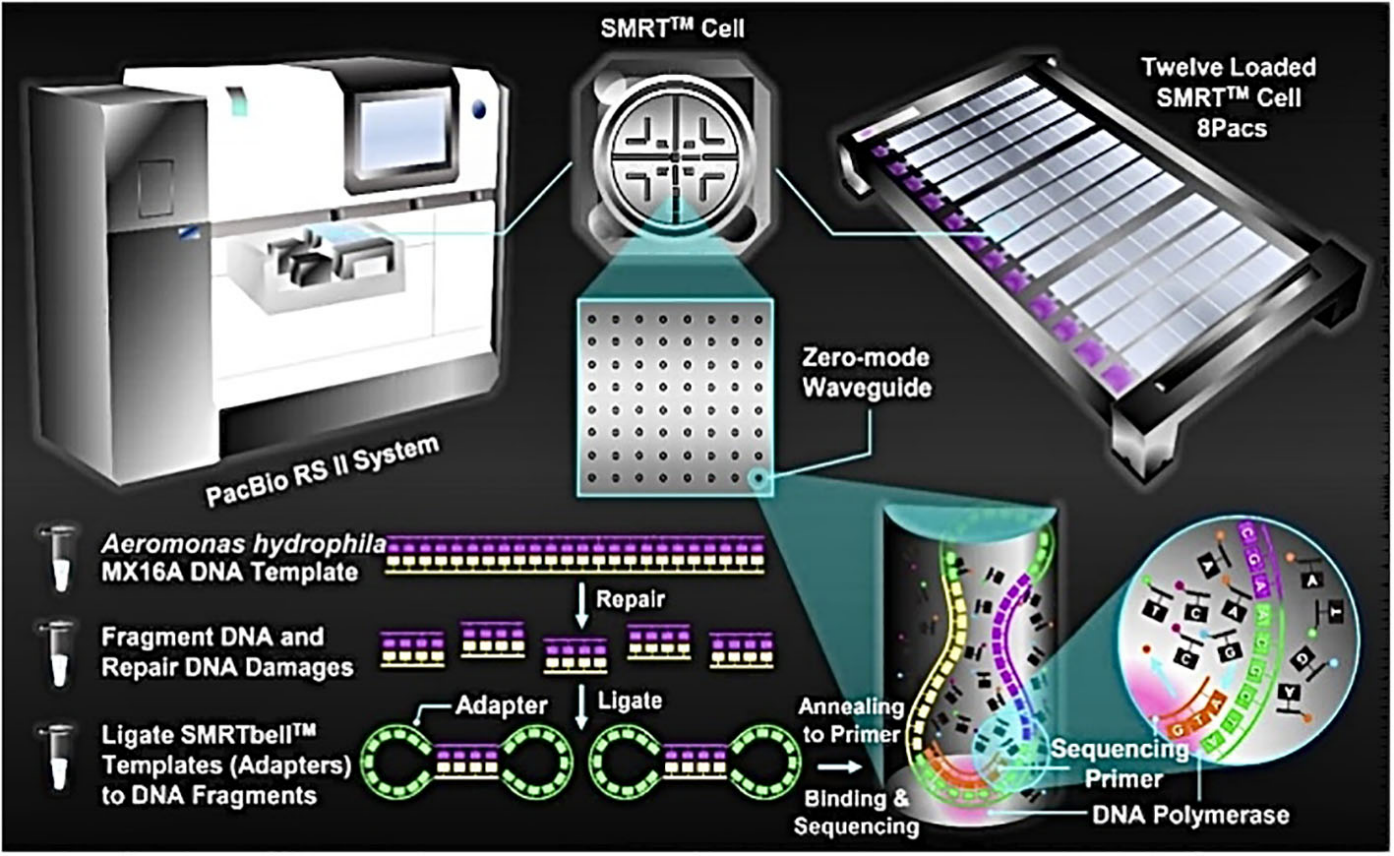
Schematic representation of PacBio’s DNA sequencing. *A. hydrophila* MX16A DNA sample was extracted by using a TIANamp Bacteria DNA Kit (Tiangen Biotech, Beijing, China) following the manufacturer’s instructions.

High-quality DNA was sent to Majorbio Company (Shanghai, China) for sequencing by PacBio RS II, a single molecule real time (SMRT™) DNA sequencing technology (Pacific Biosciences, Menlo Park, USA). Briefly, *A. hydrophila* MX16A genome DNA was first randomly sheared into 8-10 kb fragments. After repairing the damage and ends of the fragmented DNA, some appropriately sized double-standard DNA fragments called SMRTbell™ (structurally linear and topologically circular), capped by hairpin loops at both ends of the fragmented pieces. Next, a primer and a polymerase are annealed to the adapter, and the annealed templates were bound to DNA polymerase located at the bottom of zero-mode waveguides (ZMWs) in SMRT™ Cell (single molecule real time reaction pore, one SMRT™ Cell included 150 thousand ZMWs) (Ardui et al., 2018). Finally, the MX16A plate was set up for sequencing. This PacBio TGS sequencing procedure has been previously described and is shown in **Figure 1**.

### Gene prediction, rapid annotation, and data analysis

Gene prediction was carried out using Glimmer v3.02 while rRNA- and tRNA-encoding of genes was predicted using Barrnap v0.4.2 and tRNAscan-SE v1.3.1. Gene annotation and statistics were performed using blastp (BLAST 2.2.28+) and compared to the Non-Redundant (Nr) Protein Sequence Database (https://blast.ncbi.nlm.nih.gov/), Genes Database (https://www.genome.jp/kegg/genes.html/), String Database (http://string-db.org/), and Gene Ontology (GO) Database (http://www.gene-ontology.org/). Analysis of antibiotic-related genes of the *A. hydrophila* MX16A genome was performed using the Comprehensive Antibiotic Resistance Database (CARD, https://card.mcmaster.ca/). A circular map based on the bacterial chromosomal genome was generated using Circos v0.64 in Perl environment.

### Genbank accession number of MX16A’s genome

The genome sequence of *A. hydrophila* strain MX16A has been deposited in GenBank and may be found using accession number CP018201.1.

### Genome data

The genome of *A. hydrophila* was obtained from the publicly available genome database in the National Center for Biotechnology Information (NCBI). A total of 35 genomic sequences were analyzed in a comparative fashion throughout this study. The sequences were statistically classified by the host and source, including human, fish, or other hosts isolated from China, Asia (except China), South America, North America, Africa or Europe, respectively.

### Determination of average nucleotide identity

To avoid any possible genomic misidentification, all genomes were subjected to ANI check using the web based ANI calculator available at http://enve-omics.ce.gatech.edu/ani/index. In the ANI calculation, the strain *A. hydrophila* MX16A was used as a standard to analyze the genetic relationship between the *A. hydrophila* MX16A genome and the downloaded reference genome.

### Analysis of pan-genome and core-genome

Generated faa files of 33 strains were used from the Prokaryotic Dynamic Programming Genefinding Algorithm (Prodigal). These served as input files for the Bacterial Pan Genome Analysis tool (BPGA) to perform a core-pan-genome analysis. USEARCH clustering algorithm was used as parameters to run BPGA. The identity value was 0.5 and the number of combinations was 500.

### Prediction of drug resistance genes and virulence genes

Prediction of drug resistance genes and virulence genes was re-performed for 35 genomic sequences. Gff files of 35 strains were generated by Prokka. Drug resistance genes were predicted by AMRfinder (https://www.ncbi.nlm.nih.gov/pathogens/antimicrobial-resistance/AMRFinder/), and virulence factors were identified based on the core data set of the VFDB (http://www.mgc.ac.cn-/VFs/). The identity threshold used was 90% and the coverage threshold was 60%.

## Results

### Strain identification and antibiotic susceptibility

A total of 16S rRNA gene sequences clearly identified the isolate as *A. hydrophila*. Antimicrobial susceptibility testing was carried out based on the Kirby-Bauer method (**Table 1**). *A. hydrophila* MX16A was proved to be resistant to most antibiotics tested, including cefotaxime, amoxicilli, trimethoprim-sulfamethoxazole, rifampicin, erythromycin, tetracycline, streptomycin and chloramphenicol which represent β-lactams, macrolides, rifamycins, tetracyclines, sulfonamides, aminoglycosides, and chloramphenicols respectively. Based on the drug sensitivity test, it can be concluded that *A. hydrophila* MX16A was indeed a multidrug-resistant bacterium.

**Table 1:** Antimicrobial susceptibility profile of *A. hydrophila* MX16A.

Antimicrobial class	Antimicrobial agent	Disk content (μg)	Inhibition zone diameter (mm)	Interpretive Criteria			Susceptibility
				R	I	S	
β-lactams	Cefotaxime	30	14	≤22	15-22	≥26	R
	Amoxicillin	10	0	≤13	14-17	≥17	R
Quinolones	Nalidixic acid	30	11.5	≤13	14-18	≥19	R
	Norfloxacin	10	18	≤12	13-16	≥17	S
	Enrofloxacin	5	18	≤15	16-20	≥21	I
Sulfonamides	Trimethoprim- Sulfamethoxazole	1.25/23.75	0	≤10	11-15	≥16	R
Rifamycins	Rifampicin	5	13	≤16	17-19	≥20	R
Macrolides	Erythromycin	15	0	≤13	14-22	≥23	R
Tetracyclines	Tetracycline	30	10	≤11	12-14	≥15	R
Aminoglycosides	Streptomycin	10	11	≤11	12-14	≥15	R
Chloramphenicols	Chloramphenicol	30	12	≤12	13-17	≥18	R

S, susceptible; I, intermediate; R, resistant. The results were interpreted based on the guidelines by the Clinical and Laboratory Standards Institute (CLSI).

### Whole genome analysis

**Figure 2:**
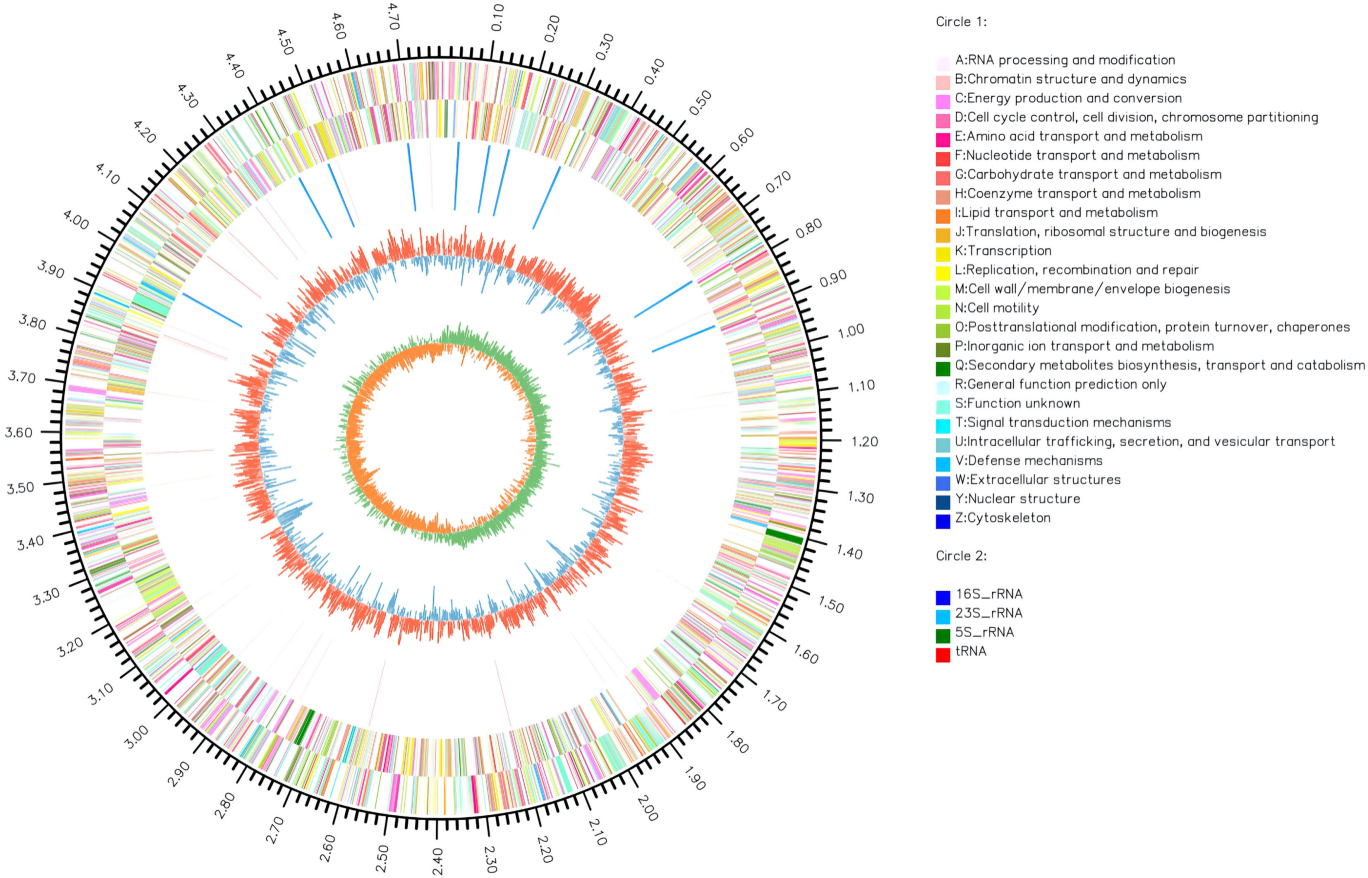
Circos circular map of *A. hydrophila* MX16A genome. The outer circle indicates the size of the genome, every scale represents 0.5 Mb; The second and third circles represent the CDS from positive-sense and negative-sense strand of genome sequences, different colors represent various functions of COG; The fourth circle represents tRNA and rRNA; The fifth circle shows the (G+C) content, the outer red portion indicates that the GC content of the region is higher than the average GC content of the whole genome; higher peaks indicate a larger difference with the average GC content; the inner blue signals indicate that the GC content of the region was lower than the average GC content of the whole genome; higher peaks indicate a larger difference with the average GC content; the innermost circle shows the GC-Skew value. The innermost circle features the GC-Skew value, the specific algorithm was (G-C)/(G+C), useful to determine the leading strand and lagging strand. In general, the leading strand GC features skew>0, the lagging strand GC features skew<0. This may further help to determine the replication starting point and the cumulative offset may also assist in determining the starting point, i.e. cumulative offset minimum, and the end point, i.e. cumulative offset maximum, of replication, particularly important for cyclic genomes.

**Figure 3:**
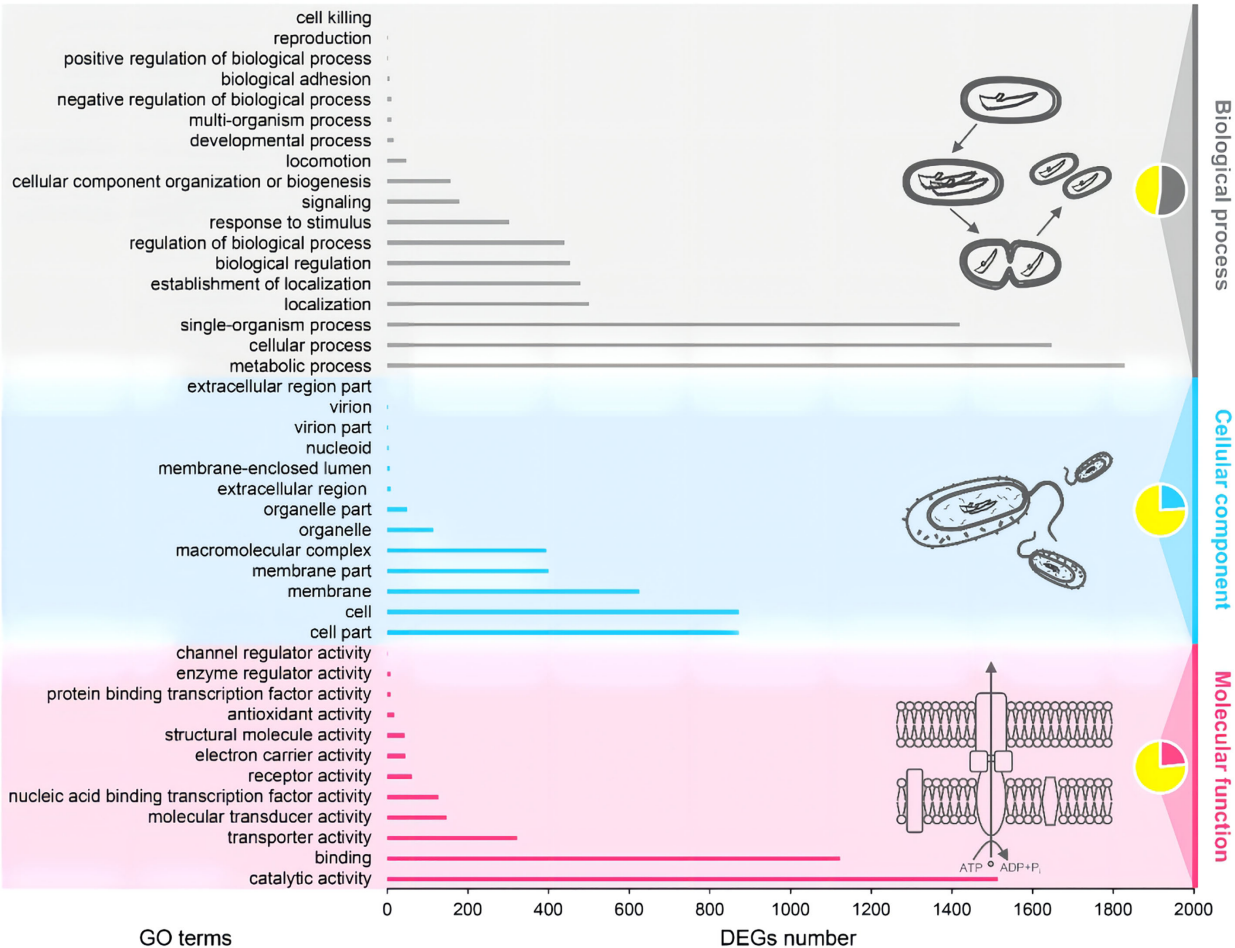
Gene ontology (GO) functional classification of *A. hydrophila* MX16A. DEGs, Differentially expressed genes.

The complete genome of *A. hydrophila* MX16A consisted of a single circular 4,168,374 bp chromosome with 4418 genes and no plasmid. The general genome features included the genomic GC content, intergeneric region length, and the number of RNA genes (**Table 2**). The circular genome map was consistent with the characteristics of bacterial genome (**Figure 2**). Furthermore, we performed a GO analysis to determine the functional classification of differentially expressed genes (DEGs) in the regulation process of MX16A. All DGEs could be combined into three main GO categories, namely biological process, cellular component, and molecular function, along with 43 subcategories as shown in **Figure 3**.

**Table 2:** General genome features of *A. hydrophila* MX16A.

Feature	Value
Gene total length (bp)	4,168,374
Intergenetic region length (bp)	615,130
GC content (%)	61.57
Gene/Genome (%)	87.10
No. of Genes	4418
No. of rRNA	31
No. of tRNA	126

### Types and distribution of resistance-associated genes

**Figure 4:**
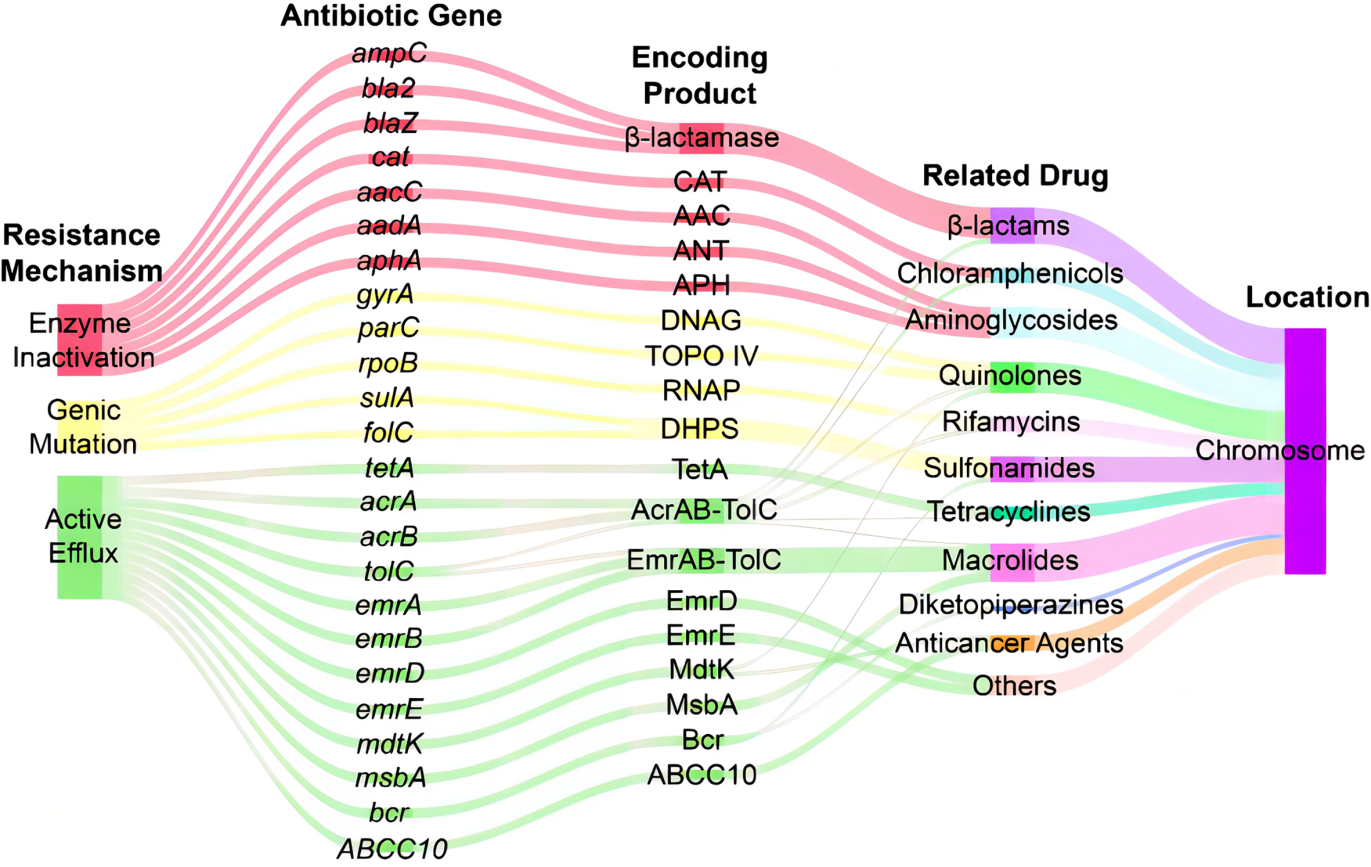
Sankey diagram of distribution of resistance-associated genes in *A. hydrophila* MX16A. CAT, chloramphenicol acetyltransferase; AAC, acetyltransferase; ANT, adenylyltransferase; APH, phosphotransferase; DNAG, DNA gyrase; TOPO IV, topoisomerase IV; RNAP, RNA polymerase; DHPS, dihydropteroate synthase.

Analysis of antibiotic-related genes of *A. hydrophila* MX16A associated with resistance to β-lactams (*ampC, bla2, blaZ*), tetracyclines (*tetA*), quinolones (*gyrA, parC*), sulfonamides (*sulA, folC*), aminoglycosides (*aphA, aacC, aadA*), chloramphenicols (*cat*), macrolides (*msbA*), and rifamycins (*rpoB*) was performed using CARD databases. Some other efflux-associated genes following by *acrA, acrB, emrA, emrB, emrD, emrE, mdtK, tolC, bcr*, and *ABCC10* (gene symbol of efflux protein MRP7) were also identified (**Table 3** and **Figure 4**).

**Table 3:** Resistance genes of the *A. hydrophila* MX16A genome extracted by CARD and Nr analysis.

Type	Antibiotic resistanceontology	Hit length(bp)	Description
β-lactams	*ampC*	382	AmpC is a class C β-lactamase, commonly isolated from extended- spectrum cephalosporin-resistant gram-negative bacteria.
	*bla2*	254	Bla2 is a chromosomal-encoded β-lactamase, found in *Bacillus anthracis*, exhibiting penicillin, cepha-lonsporin, and carbapenem- hydrolizing capabilities.
	*blaZ*	299	BlaZ is a class A β-lactamase responsible for penicillin resistance in *Staphylococcus aures*.
Chloramphenicols	*cat*	150	The *cat* gene is used to describe many variants of the chloramphenicol acetyltransferase (CAT) gene in a range of organisms.
Aminoglycosides	*aacC*	97	The *aacC* gene encodes forms of acetyltransferase (AAC).
	*aadA*	172	The *aadA* gene encodes forms of adenylyltransferase (ANT).
	*aphA*	236	The *aphA* gene encodes forms of phosphotransferase (APH).
Quinolones	*gyrA*	915	DNA gyrase (DNAG) is responsible for DNA supercoiling and consists of two α- and two β-subunits. Mutations in the quinolone resistance-determining regions (QRDRs) of these genes (*gyrA, gyrB, parC and parE*) result in amino acid substitutions that structurally change the target protein and, subsequently, the drug-binding affinity of the enzyme. In *E. coli*, the most common mutation site in *gyrA* is at Ser83 followed by Asp87, and similar mutation frequencies are seen at equivalent positions for *gyrA* and *parC* in other species.
	*parC*	764	ParC is a subunit of topoisomerase IV (TOPO IV) that decatenates and relaxes DNA to allow access to genes for transcription or translation. Point mutations in ParC prevent fluoroquinolone antibiotics from inhibiting DNA synthesis and confer low-level resistance.
Rifamycins	*rpoB*	1342	RNA polymerase (RNAP) is a multi-subunit enzyme necessary for transcription. The β-subunit of RNAP forms the active center of the enzyme and template/transcript binding sites. Mutations in *rpoB* gene confers antibiotic resistance.
Sulfonamides	*sulA*	162	The *sulA* gene encodes forms of dihydropteroate synthase (DHPS) that confer resistance to sulfonamide.
	*folC*	415	Similar to *sulA*.
Antibiotic Efflux	*tetA*	424	TetA is a tetracycline efflux pump found in various species of gram-negative bacteria.
	*acrA*	349	Protein subunit of AcrAB-TolC multidrug efflux complex. AcrA represents the periplasmic portion of the transport protein.
	*acrB*	1065	Protein subunit of AcrAB-TolC multidrug efflux complex. AcrB functions as a heterotrimer forming the inner membrane component and is primarily responsible for substrate recognition and energy transduction by acting as a drug/proton antiporter.
	*tolC*	441	TolC is a protein subunit of numerous multidrug efflux complexes in gram-negative bacteria, functions as an outer membrane efflux protein, and is constitutively open. Regulation of efflux activity often takes place at its periplasmic entrance by other components of the efflux complex.
	*emrA*	350	EmrA is a membrane fusion protein, providing an efflux pathway with EmrB and TolC between the inner and the outer membranes of *E.coli*.
	*emrB*	517	EmrB is a translocase in the EmrAB-TolC efflux protein in *E.coli*. It recognizes substrates including carbonyl cyanide m-chlorophenylhydrazone (CC-CP), nalidixic acid, and thioloactomycin.
	*emrD*	399	EmrD is a multidrug transporter from the major facilitator (MF) superfamily primarily found in *E.coli*. EmrD couples efflux of amphipathic compounds with proton import across the plasma membrane.
	*emrE*	137	EmrE is a small multidrug transporter that functions as a homodimer and couples the efflux of small polyaromatic cations from the cell with the import of protons down an electrochemical gradient. EmrE is found in *E.coli* and *Pseudomonas aeruginosa*.
	*mdtK*	440	MdtK is a multidrug and toxic compound extrusions (MATE) family transporter conferring resistance to norfloxacin, doxorubicin, and acriflavine.
	*msbA*	589	MsbA is a multidrug-resistance (MDR) transporter homolog from *E.coli* belonging to the adenosine triphosphate (ATP) binding cassette (ABC) superfamily. MsbA transports lipid A, a major component of the bacterial outer cell membrane, and is the only bacterial ABC transporter that is essential for cell viability.
	*bcr*	395	Bcr is a transmembrane protein that expels bicyclomycin from the cell, leading to bicyclomycin resistance.
	*ABCC10*	1186	ABCC10 is a ABC superfamily human protein that can transport amphipathic anions and confer resistance to several anticancer agents such as docetaxel and vincristine.

However, antimicrobial susceptibility test (**Table 1**) revealed that MX16A was not resistant to enrofloxacin and norfloxacin. We further compared the common mutation sites on the quinolone resistance- determining regions (QRDR) of *gyrA* and *parC* genes and found that Ser83 on *gyrA* and *Ser87* on parC were both mutated to Ile. For this reason, MX16A was performed additional drug sensitivity tests to nalidixic acid and found to be resistant to it.

### Overview of the genomes for comparison

The complete genomes of 35 Aeromonas hydrophila strains isolated from different regions and hosts on NCBI, including MX16A, were downloaded for genomic comparison (**Table 4**).

**Table 4:** Genomes information of 35 srains of *A. hydrophila* used in the present study.

Strain name	GenBank assembly accession	Host	Region
MX16A	GCA_001895965.1	water	China
WCHAH045096	GCA_002850695.3	water	China
NUITM-VA1	GCA_021654355.1	water	Asia (excluding China)
M052	GCA_001756325.1	water	Asia (excluding China)
GSH8-2	GCA_004296435.1	water	Asia (excluding China)
WP7-S18-ESBL-06	GCA_014161955.1	water	Asia (excluding China)
CS USB2	GCA_020162255.1	water	North America
GTCBM_22	GCA_019348635.1	water	South America
BB1457	GCA_903684605.1	water	Africa
KLG1	GCA_901212375.1	water	Europe
ZYAH72	GCA_003491225.1	fish	China
LHW39	GCA_011602425.1	fish	China
JBN2301	GCA_001455365.1	fish	China
D4	GCA_001518775.1	fish	China
B11	GCA_013205705.1	fish	China
GYK1	GCA_001683535.1	fish	China
HX-3	GCA_009791455.1	fish	China
AH10	GCA_000963645.1	fish	China
LP0103	GCA_022557195.1	fish	China
3019	GCA_018802385.1	fish	North America
3019	GCA_018802385.1	fish	North America
Aer_OnP4.2	GCA_017310115.1	fish	South America
AC185	GCA_022631175.1	fish	Asia (excluding China)
ATCC 7966	GCA_000014805.1	other	Europe
AFG_SD03_1510_Ahy_093	GCA_003323285.1	other	Asia (excluding China)
WCX23	GCA_004684305.1	other	China
RIT668	GCA_012641195.1	other	North America
S73-1	GCA_017315485.1	other	North America
A34a	GCA_015353175.1	other	Africa
ZYAH75	GCA_003491245.1	*Homo sapiens*	China
Ah2111	GCA_022982835.1	*Homo sapiens*	China
CN17A0135	GCA_016729115.1	*Homo sapiens*	China
AHNIH1	GCA_001687125.1	*Homo sapiens*	North America
RIMD111065	GCA_016592295.1	*Homo sapiens*	Asia (excluding China)
Aer284	GCA_003849735.1	*Homo sapiens*	South America
S-P-C-021.01	GCA_022488365.1	*Homo sapiens*	Africa

### Comparative analysis of average nucleotide identity

**Figure 5:**
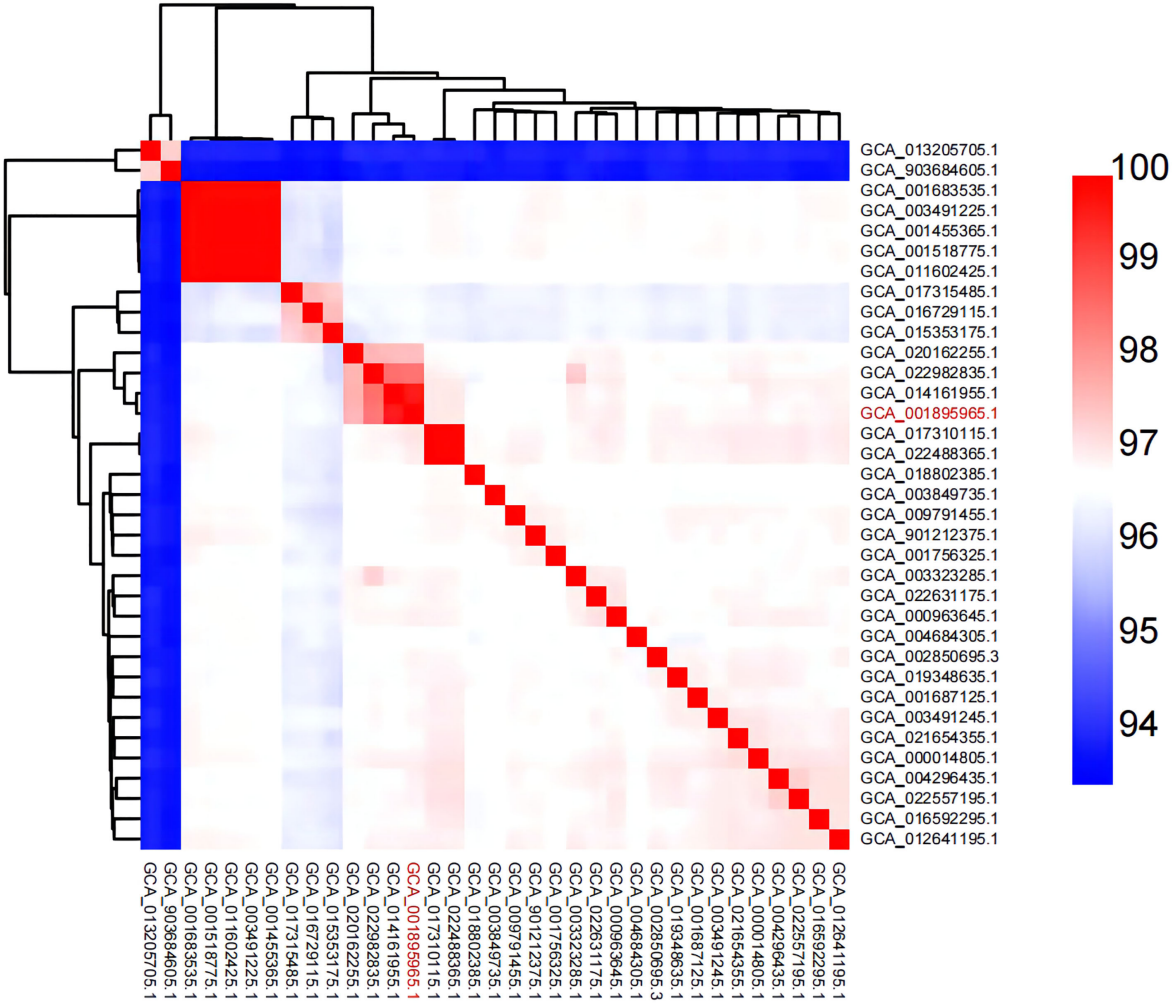
Heat map of ANI pairings between any two genomes of MX16A (in red font) and other *A. hydrophila* strains downloaded from NCBI database.

In recent studies, whole-genome average nucleotide identity (ANI) has emerged as a robust method for assessing species boundaries. Typically, organisms belonging to the same species have ≥95% ANI values. ANI represents the average nucleotide identity of all orthologous genes shared between any two genomes and offers robust resolution between strains of the same or closely related species (showing 80–100% ANI) (Jain et al., 2010). We performed ANI analysis on the whole genome of 35 Aeromonas hydrophila strains used in this study (**Figure 5**; **Supplementary Table 1**). The figure shows a region with high similarity and a higher genomic diversity. The analysis revealed that strains GCA_014161955.1 and GCA_022982835.1 featured high similarity with *A. hydrophila* MX16A. The corresponding ANI was 99.6% and 98.6%, respectively. The similarity of strains GCA_013205705.1 and GCA_903684605.1 with MX16A was below 95%. This finding may indicate that these two strains were misclassified as *A. hydrophila.*

### Comparative genome analysis

**Figure 6:**
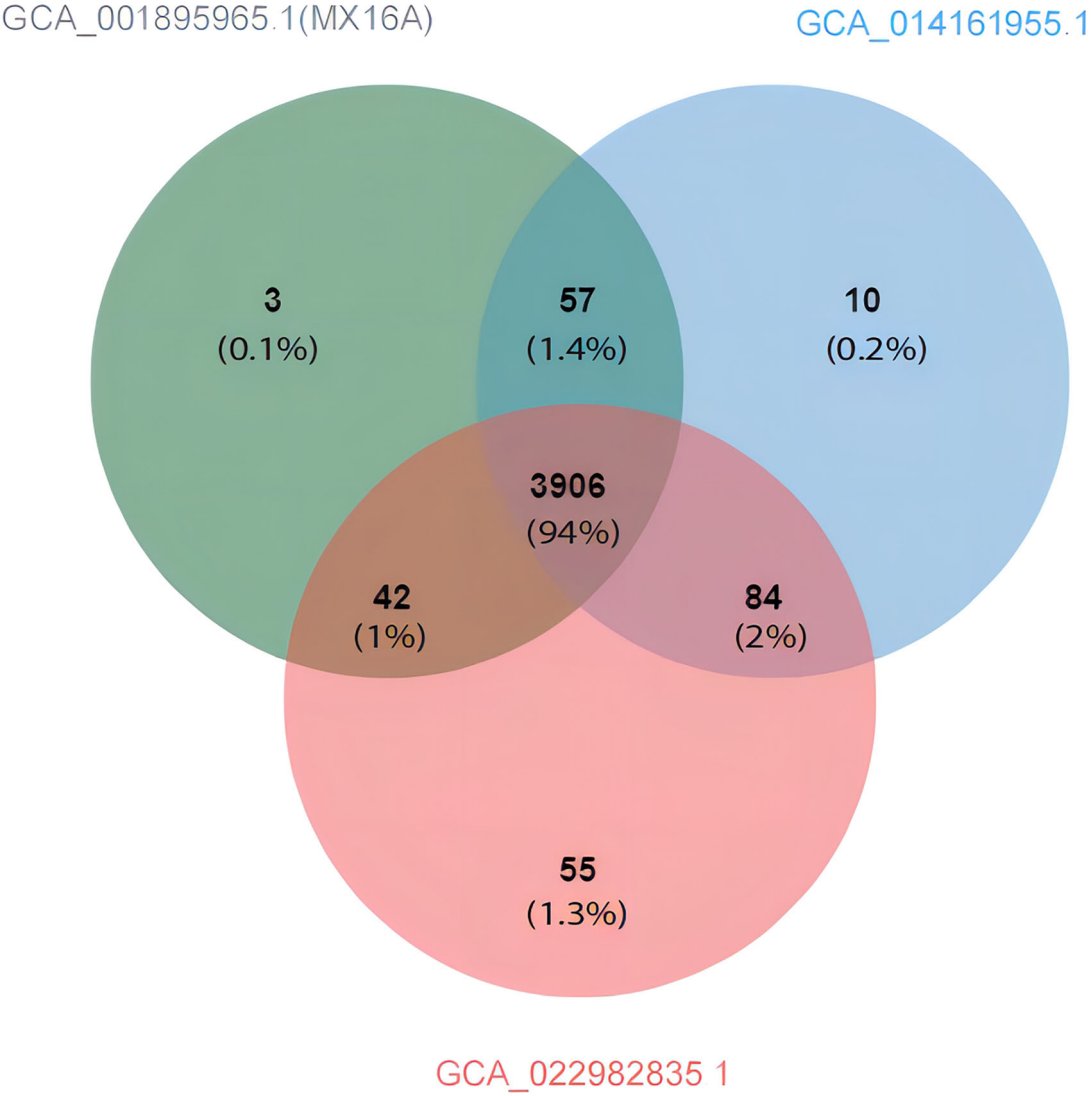
Venn diagram showing the protein shared and exclusive among *A. hydrophila* GCA_001895965.1 (MX16A), GCA_014161955.1, and GCA_022982835.1.

Compared with *A. hydrophila* MX16A, only GCA_014161955.1 and GCA_022982835.1 featured greater ANI than 98%. Using the genomes of the three strains as inputs, OrthoFinder was used to pally align the protein sequences of the strains and detect the shared protein between the strains. Based on the generated files (Orthogroups. GeneCount. TSV) for processing, the protein contained in each of the three genomes were sorted out. Jvenn was used to draw a comparative analysis diagram of the clustering results of the three strains protein (**Figure 6**). The results obtained were similar to the ANI prediction and strain MX16A was demonstrated to be more similar to GCA_014161955.1 as these two strains shared most of the protein. Strain GCA_022982835.1 featured a larger number of unique proteins.

### Pan-core genome analysis

**Figure 7:**
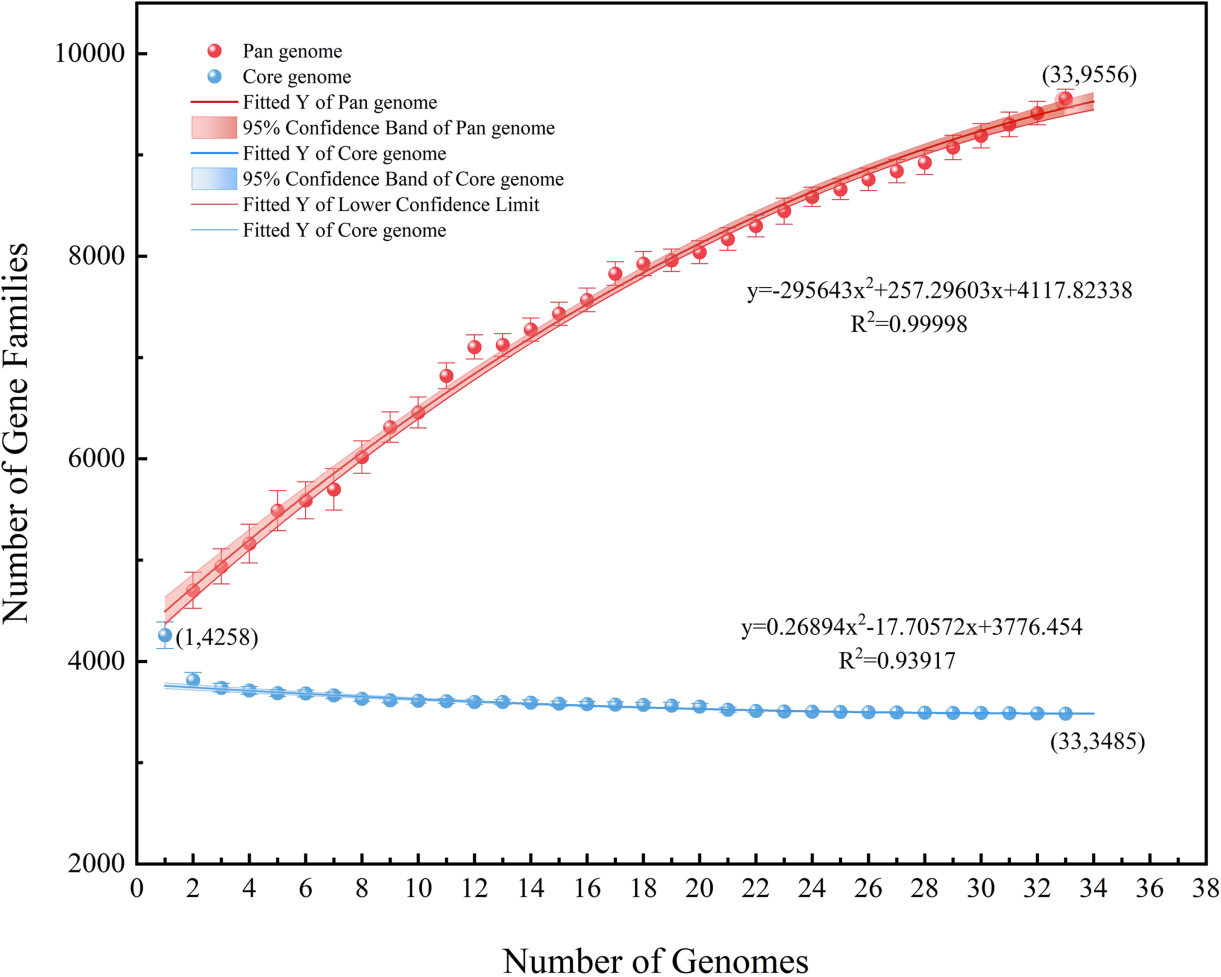
Pan-core genome analysis of 33 strains of *A. hydrophila*, except the two possibly misclassified strains (ANI<95%).

Excluding the two strains with an ANI lower than 95%, the remaining 33 strains were analyzed regarding their pan-core genome. As the number of genes increased, the number of core genes gradually decreased and was then found to plateau while the pan-genome continued to expand. The final core genome size was converged to 3485 genes and the final pan-genome was converged to 9556 genes (**Supplementary Tables 2**, **3**), presenting an open state (**Figure 7**). It may be speculated in this context that the *A. hydrophila* genome featured a more stable core gene cluster with high partial genome variability. These highly variable sections of the genome potentially enable different environmental adaptations.

### Analysis of drug resistance and virulence factors

**Figure 8:**
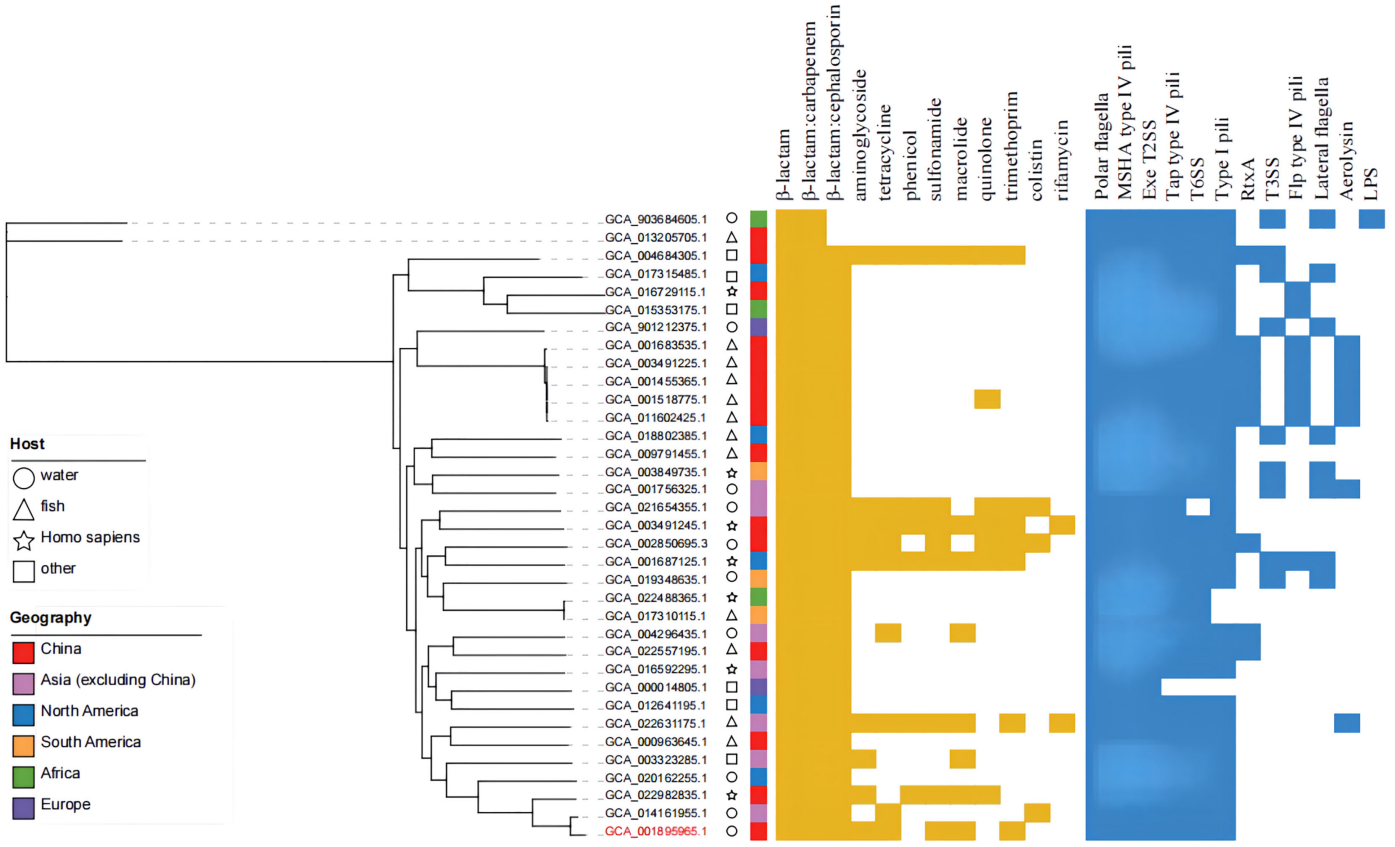
Phylogenetic tree, distribution of drug-resistance genes and virulence factors of 35 strains of *A. hydrophila*.

The online tool ITOL (https://itol.embl.de/) was used to generate evolutionary trees and maps of drug resistance genes and virulence factors (**Figure 8**). A total of 12 classes of resistance genes were predicted in these genomes, of which β-lactam and β-lactam:carbapenem genes were shared by 35 strains. Genes of β-lactam:cephalosporin were also shared by alomost all strains, except the two possibly misclassified strains GCA_013205705.1 and GCA_903684605.1 (ANI<95%). Strain MX16A featured a larger number of resistance genes (**Supplementary Table 4)**. For virulence factors, 6 classes of virulence factors could be determined, common to most strains. The strains GCA_003491225.1, GCA_001455365.1, GCA_001518775.1, GCA_001683535.1 and GCA_011602425.1 exhibited highly similar drug resistance genes and virulence factors, all isolated from China with fish being the host. However, these strains stem from different Chinese provinces such as Guangdong and Hubei, potentially resulting in spreading due to breeding and transportation.

## Discussion

### Drug resistance and resistance-associated genes of *A. hydrophila* MX16A

From antibiotic sensitivity tests. we could determine that *A. hydrophila* MX16A was resistant to all clinically used drugs except for some quinolones. This finding was consistent with the results of the majority of drug resistance genes predicted by CARD.

### Enzyme mediated drug resistance

β-lactam antibiotics have been extensively used to against gram-negative pathogens due to their broad spectrum of antibacterial activity these drugs exhibit until the appearance of the β-lactam inactivator β-lactamase (Abraham and Chain, 1988). The latter enzymes hydrolyze the β-lactam ring and therefore reduce the overall activity of β-lactam drugs (Davies, 1994). The process of enzymatic hydrolization is regarded as the primary resistance mechanism to β-lactam antibiotics in drug-resistant pathogens. Overexpression of various chromosomally-mediated-β-lactamases in *A. hydrophila* is generally associated with β-lactam as reported previously (Ko et al., 1998; Saavedra et al., 2004; Balsalobre et al., 2009). As such, the chromosome-borne enzyme AmpC coded as gene *ampC* has been found to inactivate carbapenem antibiotics in *Enterobacteriaceae* bacteria (Suh et al., 2010) while the β-lactamase Bla2, coded as chromosomal gene *bla2*, has been found to be expressed in *Escherichia coli*, resulting in β-lactam hydrolyzation and drug resistance against antibiotics based on carbapenem, cephalosporinase, and penicillinase (Materon et al., 2003).

Chloramphenicol acetyltransferase (CAT), encoded by the gene *cat*, represents the main factor for chloramphenicol resistance demonstrated by some bacteria strains. This type of antibiotic inactivating enzyme may catalyze the acetyl-S-CoA-dependent acetylation of chloramphenicol at the 3-hydroxyl group to transfer chloramphenicol to a metabolite without antimicrobial activity (Shaw, 1983). Moreover, in bacteria, resistance to aminoglycosides is usually deemed to enzymatic inactivation by acetyltransferases (encoded by gene *aac*), adenylyltransferases (encoded by gene *aad*), and phosphotransferases (encoded by gene *aph*) (Shaw et al., 1993).

### Genetic mutation-mediated drug resistance

Naturally occurring resistance to fluoroquinolones in gram-negative bacteria is usually due to the genetic mutation of DNA gyrase (encoded by *gyrA*) and topoisomerase IV (encoded by *parC*), both of which represent the enzyme targets of fluoroquinolones in the bacterial cell and play a key role in DNA replication (Drlica and Zhao, 1997; Hooper, 2000). These enzymatic changes in the target site may block the formation of compounds by these enzymes and fluoroquinolones, usually responsible for bacterial DNA replication inhibition (Hooper, 2000). Furthermore, the region for mutations within *gyrA* and *parC* has been described as the quinolone-resistance determining region (QRDR) (Yoshida et al., 1990) and is associated with the mechanism of quinolone-resistance in *Aeromonas* spp. as reported previously (Alcaide et al., 2010). Moreover, anti-rifampicin strains have been reported with alteration of the *rpoB* gene encoding the beta subunit of RNA polymerase. A large majority of rifampicin-resistant isolates including Aeromonas strains are related to specific mutations within the 81-bp region (rifampicin-resistance determining region, RRDR) of the *rpoB* gene as reported in the literature (Herrera et al., 2003; Zhou et al., 2012; Pridgeon et al., 2013).

For living cells, folate derivatives, synthesized by most bacterial species, generally represent the requisite cofactors participating in the biosynthesis of purines, pyrimidines, and amino acids. Thus, enzymes in the pathway of folate biosynthesis represent the paramount targets to folate-inhibitors, including sulfonamides, to antagonize pathogens (Goulding et al., 2005). Earlier reports have revealed that sulfonamide-resistance was closely associated with genetic mutation of the dihydropteroate synthase (DHPS) encoded by the chromosomal gene *sul* and *fol* (e.g. *sulA* and *folC*) in bacteria (Maskell et al., 1997; Swedberg and Ringertz, 1998; Haasum et al., 2001).

### Efflux protein-mediated drug resistance

Efflux pumps are one of the major causes of MDR in almost all bacterial species with gene-encoding proteins found on the chromosomes or plasmids (Webber and Piddock, 2003; Poole, 2007). According to the structural diversity, energy sources, and substrates, efflux pumps of gram-negative bacteria can be divided into five families: ATP-binding cassette (ABC) superfamily, major facilitator (MF) superfamily, resistance nodulation division (RND) family, multi-antimicrobial extrusion (MATE) family, and small multidrug-resistance (SMR) family (Blair et al., 2014).

As members of the ABC superfamily proteins, MRP7/ABCC10 (gene symbol *ABCC10*) may transport amphipathic anions and cause resistance to several anticancer agents such as docetaxel and vincristine (Hopperborge et al., 2004; Kruh et al., 2007) while MsbA protein (encoded by gene *msbA* and localized to the inner membrane) was found to cause resistance to erythromycin by means of ATP binding and hydrolytic properties in both gram-positive (Woebking et al., 2005) and gram-negative (Ghanei et al., 2007) microorganisms. The corresponding gene *tetA* located on the chromosome encoding TetA efflux protein (generally associated with MF superfamily) frequently observed in *Aeromonas* spp. isolates (Jacobs and Chenia, 2007) has been described to leave the host bacterium unharmed by actively transporting intracellular tetracycline out of cell cytoplasm (Roberts, 2005; Wang et al., 2014). Previous studies have revealed that overproduction of MdtK, a MATE family membrane transport protein, encoded by gene *mdtK*, could induce resistance to norfloxacin, doxorubicin, and acriflavine in *Salmonella enterica* (Horiyama et al., 2010).

TolC (encoded by *tolC*), an outer membrane protein, functions as the outer membrane channel to several MDR efflux systems (Benz et al., 1993), including the AcrAB-TolC (RND-type) and EmrAB-TolC (MF-type). AcrAB-TolC represents the main efflux system commonly found in various gram-negative bacteria (Pradel and Pagès, 2002; Baucheron et al., 2004; de Cristóbal et al., 2006), forming by a tripartite complex comprised of a cytosolic membrane pump (AcrB, encoded by acrB), a periplasmic membrane fusion protein (AcrA, encoded by *acrA*), and a TolC protein (Du et al., 2014). The EmrAB-TolC efflux system is also composed of three componentes including the membrane protein EmrA (encoded by *emrA*), transporter protein EmrB (encoded by *emrB*), and outer membrane protein TolC (Lomovskaya and Lewis, 1992). These two above-mentioned efflux systems, particularly AcrAB-TolC, have been reported to significantly affect the antimicrobial resistance to multiple antibiotic classes such as tetracyclines, chloramphenicols, rifamicins, β-lactams, quinolones, and macrolides in gram-native bacteria (Okusu et al., 1996; Nishino et al., 2003; Baucheron et al., 2004; Chollet et al., 2004; Elkins and Mullis, 2007). Moreover, EmrD (MF superfamily) and EmrE (SMR family) represent two membrane transporter proteins codified by genes *emrD* and *emrE*, respectively. Both of these proteins may induce drug resistance by involving adaptation to low energy shock or exporting diverse drug substrates protecting bacteria from antibiotic damage according to various studies reported in the literature (Naroditskaya et al., 1993; Rotem and Schuldiner, 2004). Moreover, gene *bcr* encoding a drug resistance bacterial transmembrane protein (Bcr), was also identified from the *A. hydrophila* MX16A genome involved in bicyclomycin and sulfathiazole resistance in *E. coli* (Bentley et al., 1993; Hayashi et al., 2010).

### Comparative genome analysis

As described above, we were able to assess the diversity level of the biological genome to some extent related to the ratio of core genome to pangenome. The pangenome analysis of *A. hydrophila* described in this paper showed a large genomic diversity and further demonstrated that this species could adapt to and survive in different environmental conditions. In a previous study found in the literature (Ghatak et al., 2016), the pan-genome and core genes of 16 strains of *Aeromonas* in the genus *Aeromonas* spp. were analyzed and the results showed that *A. hydrophila* exhibited a higher genome diversity than *A. veronii* and *A. caviae*. Combined with our analytical data, the number of genes in the core genome did not change significantly, further confirming that the core genome of *A. hydrophila* is indeed stable. Finally, it may also be speculated that the genome of *A. hydrophila* features rich metabolic functions.

### Analysis of virulence and drug resistance factors

Type III secretion systems are considered an important virulence mechanism for aeromonads (Sierra et al., 2010; Krzymińska et al., 2012). In this study, we did not predict genes for the type III virulence system, however, Ghatak et al., reported genes related to the type III secretion systems are present in all genomes of *A. hydrophila*, presumably due to the use of different databases. Importantly, a different drug resistance gene database for *A. hydrophila* MX16A was used in this report, resulting in some differences in the forecasted results. Several strains with more and roughly similar resistance genes stemed from different host types and were distributed in different regions. Due to the wide geographical distribution of *A. hydrophila* and the wide distribution of its hosts, it is hypothesized that frequent gene exchange between strains may lead to the transfer of corresponding drug resistance genes.

## Conclusions

In this study, we report preliminary results regarding the environmental MDR *Aeromonas hydrophila* strain MX16A based on complete genome sequence analysis. The results of resistance gene prediction analysis were mostly consistent with the phenotypic antimicrobial susceptibility test. A variety of antibiotic-related genes identified from the *A. hydrophila* MX16A genome indicates that this bacterium may pose a potential threat to both human and aquatic animal health with phylogenetic analysis further confirming this conclusion. The diverse types and distributions of antibiotic genes of *A. hydrophila* MX16A suggested that the bacterial resistance mechanism was variable as follows: enzyme inactivation, genetic mutation, and active efflux. However, it still remains unclear which and how these three variables influence the overall drug resistance mechanism of *A. hydrophila* MX16A. Thus, future studies may now focus on the genomic characteristics potentially further leading to the development of targeted antibiotics. *A. hydrophila* MX16A exhibited close similarities to GCA_022982835.1. Due to the finding that humans are the primary host for the GCA_022982835.1 strain, it may be concluded that the pathway of human infection with *Aeromonas* may be due to the presence of this bacterial species in water and infected aquatic animals. Genome comparison studies carried out throughout this research further revealed that *A. hydrophila* displays a wide host and geographic distribution pattern, is not conserved, features high genome variability, and horizontal gene transfer may promote the evolution of other strains.

## Data availability statement

The datasets presented in this study can be found in online repositories. The names of the repository/repositories and accession number(s) can be found in the article/**Supplementary Material.**

## Author contributions

YG and CZ carried out the experiments and drafted the manuscript. ML directed the research and revised the manuscript. CM isolated the bacterial strain. HC, XJ, SZ, and XX analyzed the genomic data and revised the manuscript. All authors contributed to the article and approved the submitted version.

## Funding

This research was supported by the National Key R&D Program of China (2020YFD0900102), the Earmarked Fund for China Agriculture Research System (No.CARS-46), the Xiamen Ocean and Fishery Development Special Fund (21CZP007HJ07).

## Conflict of interest

The authors declare that the research was conducted in the absence of any commercial or financial relationships that could be construed as a potential conflict of interest.

## Publisher’s note

All claims expressed in this article are solely those of the authors and do not necessarily represent those of their affiliated organizations, or those of the publisher, the editors and the reviewers. Any product that may be evaluated in this article, or claim that may be made by its manufacturer, is not guaranteed or endorsed by the publisher.
